# Shaking the Tree: Multi-locus Sequence Typing Usurps Current Onchocercid (Filarial Nematode) Phylogeny

**DOI:** 10.1371/journal.pntd.0004233

**Published:** 2015-11-20

**Authors:** Emilie Lefoulon, Odile Bain, Jérôme Bourret, Kerstin Junker, Ricardo Guerrero, Israel Cañizales, Yuriy Kuzmin, Tri Baskoro T. Satoto, Jorge Manuel Cardenas-Callirgos, Sueli de Souza Lima, Christian Raccurt, Yasen Mutafchiev, Laurent Gavotte, Coralie Martin

**Affiliations:** 1 Unité Molécules de Communication et Adaptation des Microorganismes, Sorbonne Universités, Muséum national d’Histoire naturelle, Paris, France; 2 ARC-Onderstepoort Veterinary Institute, Onderstepoort, South Africa; 3 Laboratorio de Biologia de Vectores y Parásitos, Instituto de Zoologia y Ecologia Tropical, Facultad de Ciencias, Universidad Central de Venezuela, Caracas, Venezuela; 4 Institute of Zoology, Ukrainian National Academy of Sciences, Kiev, Ukraine; 5 Center For Tropical Medicine, Universitas Gadjah Mada, Jl Teknika Utara Yogyakarta, Indonesia; 6 Asociación Peruana de Helmintologia e Invertebrados Afines (APHIA), Peru; 7 LTEH ODILE BAIN, Departamento de Zoologia, Instituto de Ciências Biológicas Universidade Federal de Juiz de Fora, Minas Gerais, Brasil; 8 Laboratoire National de Santé Publique, Port-au-Prince, Haiti; 9 Institute of Biodiversity and Ecosystem Research, Bulgarian Academy of Sciences, Sofia, Bulgaria; 10 Université Montpellier, place Eugène Bataillon, Montpellier, France; McGill University, CANADA

## Abstract

During the past twenty years, a number of molecular analyses have been performed to determine the evolutionary relationships of Onchocercidae, a family of filarial nematodes encompassing several species of medical or veterinary importance. However, opportunities for broad taxonomic sampling have been scarce, and analyses were based mainly on 12S rDNA and *coxI* gene sequences. While being suitable for species differentiation, these mitochondrial genes cannot be used to infer phylogenetic hypotheses at higher taxonomic levels. In the present study, 48 species, representing seven of eight subfamilies within the Onchocercidae, were sampled and sequences of seven gene loci (nuclear and mitochondrial) analysed, resulting in the hitherto largest molecular phylogenetic investigation into this family. Although our data support the current hypothesis that the Oswaldofilariinae, Waltonellinae and Icosiellinae subfamilies separated early from the remaining onchocercids, Setariinae was recovered as a well separated clade. *Dirofilaria*, *Loxodontofilaria* and *Onchocerca* constituted a strongly supported clade despite belonging to different subfamilies (Onchocercinae and Dirofilariinae). Finally, the separation between Splendidofilariinae, Dirofilariinae and Onchocercinae will have to be reconsidered.

## Author Summary

Filariae are predominantly tissue-dwelling nematodes of the Onchocercidae (Spirurida). They are parasites of terrestrial vertebrates and some of them are particularly well known as agents of human diseases in tropical environments (*e*.*g*. onchocercosis, lymphatic filariosis, loaosis). Because of their predilection for host tissue sites, filariae are difficult to collect and their diversity is far from being well-investigated. Given the lack of fossilized material, it is not possible to formulate a comprehensive evolutionary hypothesis for this group based exclusively on morphological studies. Molecular analyses on the other hand have so far been based on a limited number of filarial species only or have used genetic markers convenient for species differentiation but not for retracing filarial history. Thus a consistent evolutionary framework for this family is still not in place. The originality and strength of the present study rest within its ability to access an unusually large range of biological samples, comprising 48 species representing seven of the eight onchocercid subfamilies. Using a new multi-gene dataset analysis, five major clades within the family were defined, including i) a group of ancestrally-derived subfamilies, ii) Setariinae as a sister group to all remaining subfamilies and iii) one large clade comprising genera of the Dirofilariinae, Onchocercinae and Splendidofilariinae.

## Introduction

The Onchocercidae (Spirurida), commonly referred to as filariae, are a family of parasitic nematodes characterised by a wide host-range in squamates, crocodilians, amphibians, mammals and birds [1]. As several filarial species are agents of human and veterinary diseases [2–4], the Onchocercidae have been the subject of numerous studies. Presently, the family is divided into eight subfamilies, including 88 genera [5–8]. They are nematodes with an evolved life cycle, involving blood- or skin-inhabiting first-stage larvae transmitted by haematophagous arthropods vectors [1,9].

A pronounced regression of morphological features and a number of convergences resulting from a parasitic life style, as well as the absence of fossilised material, make it difficult to produce phylogenetic hypotheses for this family [10]. Thus, major questions regarding the classification of the Onchocercidae, their origin and evolution remain as yet unresolved. In numerous morphological and biological studies an attempt has been made to elucidate the evolution of the Onchocercidae [1,10]. First drawn up by Wehr in 1935 [11], the classification of the family has been reconstructed several times [12,13], with the most comprehensive analysis being that of Anderson and Bain in 1976 [6].

In 1994 [14], the development of molecular techniques made the construction of the first molecular phylogeny of the Onchocercidae, based on the 5S rDNA sequences, possible. This pioneering study focused on ten species from six genera, representing two subfamilies (Dirofilariinae and Onchocercinae). With the rise of the DNA barcoding approach to distinguish between species, studies on filarial nematode phylogeny have been predominantly based on 12S rDNA and *coxI* gene analyses [15–18], and phylogenies for selected onchocercid species have been proposed. However, biological material has been scarce, impeding broad taxonomic sampling, and the markers used were not suitable to resolve the internal nodes which describe the evolution of the Onchocercidae [19]. Finally, a few studies have been conducted using alternative genes, but were either focused on nematodes in general [20–22], a specific genus [23,24] or based on the mitochondrial genome [25], all including a limited number of species.

The aim of the present study was to propose a robust phylogenetic hypothesis of the relationships within the Onchocercidae based on the concatenation of seven loci (two mitochondrial and five nuclear genes) of 48 belonging to seven subfamilies. This hitherto most comprehensive sampling of the Onchocercidae casts doubt on the biological validity of some of the classically defined subfamilies.

## Materials and Methods

### Material

Sixty-two specimens comprising 48 species belonging to 25 genera and representing seven of eight onchocercid subfamilies were analysed (Table 1 and S1 Table) [8,9,26–59]. Due to a lack of material, the Lemdaninae could not be included in this study. All procedures were conducted in compliance with the rules and regulations of the respective competent national ethical bodies (S2 Table). Nematode specimens and DNA samples were deposited in the National Nematode collection of the Muséum National d’Histoire Naturelle (MNHN), Paris, France; accession numbers are recorded in Tables 1 and S1. Below we briefly list the specimens used in this study, followed by the source/collector in parentheses. For the author(s) and year of parasite and host (authors only) species, the reader is referred to Table 1 and S1 Appendix.

DNA, L3, microfilariae.

**Table 1:** List of filarial species used in phylogenetic analyses in this study. The onchocercid subfamilies are indicated in the first column. The name of the species as well as the author(s) and date are given in the second column. Columns 3 to 5 contain sample information, *i*.*e*. host species, MNHN accession number and collection locality.

Subfamilies	Species	Host	N° MNHN	Collection locality
Outgroups	*Filaria latala* Chabaud and Mohammad, 1989	*Panthera leo*	62YT	South Africa
	Protospirura muricola* Gedoelst, 1916	*Gorilla gorilla*	97YU	Central African Republic
Oswaldofilariinae	*Oswaldofilaria chabaudi Pereira*, *Souza and Bain*, *2010*	*Tropidurus torquatus*	191YU	Brasil
	*Oswaldofilaria petersi Bain and Sulahian 1974*	*Crocodilurus amazonicus*	34PF	Peru
Waltonellinae	*Ochoterenella* sp.1 Guerrero et *al*. work in progress	*Rhinella granulosa*	200YU	Venezuela
	*Ochoterenella sp*.*2 Guerrero et al*. work in progress	*Rhinella marina*	47YT	Venezuela
	*Ochoterenella sp*.*3 Kuzmin et al*. work in progress	*Phyllomedusa bicolor*	194JW	French Guyana
Icosiellinae	*Icosiella neglecta* (Diesing, 1851)	*Pelophylax ridibunda*	44YT	Ukraine
	* *	*Pelophylax kl*. *esculeta*	45YT	France
Setariinae	*Setaria labiatopapillosa* (Alessandrini, 1848)	*Bos taurus*	413YU	Cameroon
	*Setaria tundra* Bain, 1974	*Rangifer tarandus*	71YT	Finlande
Dirofilariinae	*Dirofilaria* (*Dirofilaria*) *immitis* (Leidy, 1856)	*Canis familiaris*	79YT	Bayer strain
	*Dirofilaria (Nochtiella) repens Railliet and Henry*, *1911*	*Canis familiaris*	297YU	Italy
	*Foleyella candezei* (Fraipont, 1882)	*Agama agama*	68CE	Togo
	*Loa loa* (Cobbold, 1864)	*Homo sapiens*	80YT	France
	*Pelecitus fulicaeatrae* (Diesing, 1861)	*Podiceps nigricollis*	49YT	Spain
Splendidofilariinae	*Aproctella alessandroi Bain*, *Petit*, *Kosek and Chabaud*, *1981*	*Saltator similis*	117YU	Brasil
	*Cardiofilaria pavlovskyi* Storm, 1937	*Oriolus oriolus*	180YU	Bulgaria
	*Madathamugadia hiepei* Hering-Hagenbeck, Boomker, Petit, Killick-Kendrick and Bain, 2000	*Pachycactylus turneri*	81YU	South Africa
	*Rumenfilaria andersoni Lankester and Snider*, *1982*	*Rangifer tarandus*	94YU	Finlande
Onchocercinea	*Acanthocheilonema odendhali* (Perry, 1967)	*Callorhinus ursinus*	401YU	Alaska
	*Acanthocheilonema viteae* (Krepkogorskaya, 1933)	*Meriones unguiculatus*	7YT	FR3 strain
	*Breinlia* (*Breinlia*) *jittapalapongi* Veciana, Bain, Morand, Chaisiri, Douanghoupha, Miquel and Ribas, 2015	*Rattus tanezumi*	78YT	Laos
	*Brugia malayi* (Brug, 1927)	*Meriones unguiculatus*	8YT	FR3 strain
	*Brugia pahangi (Buckley and Edeson*, *1956)*	*Meriones unguiculatus*	46YT	FR3 strain
	*Brugia timori* Partono, 1977	*Homo sapiens*	6YT	Indonesia
	*Cercopithifilaria bainae Almeida and Vicente*, *1984*	*Canis familiaris*	9YT	experimental
	*Cercopithifilaria rugosicauda (Böhm and Supperer*, *1953)*	*Capreolus capreolus*	350YU	France
	*Cruorifilaria tuberocauda Eberhard*, *Morales and Orihel*, *1976*	*Hydrochoerus hydrochaeris*	55YT	Venezuela
	*Dipetalonema caudispina* (Molin, 1858)	*Ateles paniscus*	362YU	Guyana
		*Ateles sp*.	64YT	Guyana
	*Dipetalonema gracile* (Rudolphi, 1809)	*Cebus olivaceus*	124CV	Venezuela
	* *	*Cebus apella*	215YU	Peru
	* *	*Ateles sp*.	63YT	Guyana
	*Dipetalonema graciliformis* (Freitas, 1964)	*Saimiri scuireus*	220YU	Peru
	*Dipetalonema robini Petit*, *Bain and Roussilhon*, *1985*	*Lagothrix poeppigii*	216YU	Peru
	*Litomosoides brasiliensis* Lins de Almeida, 1936	*Carollia perspicillata*	35/37PF	Peru
	*Litomosoides hamletti* Sandground, 1934	*glossophaga soricina*	36PF1	Peru
	*Litomosoides sigmodontis* Chandler, 1931	*Meriones unguiculatus*	186MS	MNHN strain
	*Litomosoides solarii* Guerrero, Martin, Gardner and Bain, 2002	*Trachops cirrhosus*	213YU	Venezuela
	Loxodontofilaria caprini Uni and Bain, 2006	*Naemorhedus crispus*	YG2-58	Japan
	Mansonella (Cutifilaria) perforata Uni, Bain and Takaoka, 2004	*Cervus nippon*	216JW	Japan
	*Mansonella* (*Mansonella*) *ozzardi* (Manson, 1897)	*Homo sapiens*	77YT	Haiti
	Monanema martini Bain, Bartlett and Petit, 1986	*Arvicanthis niloticus*	31NC	Senegal
	*Onchocerca armillata* Railliet and Henry, 1909	*Bos taurus*	54FK	Cameroon
	*Onchocerca dewittei japonica* Uni, Bain and Takaoka, 2001	*Sus scrofa leucomystax*	OB9	Japan
	*Onchocerca eberhardi* Uni and Bain, 2007	*Cervus nippon*	S63-5	Japan
	*Onchocerca gutturosa* Neumann, 1910	*Bos taurus*	54FK	Cameroon
	*Onchocerca ochengi* Bwangamoi, 1969	*Bos taurus*	54FK	Cameroon
	*Onchocerca skrjabini* Ruklyadev, 1964	*Cervus nippon*	S63-6	Japan
	*Yatesia hydrochoerus* (Yates, 1980)	*Hydrochoerus hydrochaeris*	52YT	Venezuela

*Protospirura muricola is typically a parasite of rodents, but has been reported from primates [78].

From the following species, DNA had been extracted from adult worms for previous [18,19,41] and deposited in the MNHN collection (Table 1): *Madathamugadia hiepei*, *Rumenfilaria andersoni*, *Cercopithifilaria rugosicauda*, *Aproctella alessandroi*, *Loxodontofilaria caprini*, *Mansonella* (*Cutifilaria*) *perforata*, *Onchocerca dewittei japonica*, *Onchocerca eberhardi*, *Onchocerca skrjabini* and *Ochoterenella* sp. 1. DNA of adult *Setaria labiatopapillosa* (Dr Ben Makepeace, UK) was given to us. DNA of *Cercopithifilaria bainae* had been extracted from infective larvae from *Ixodes ricinus* (Prof Domenico Otranto, Italy). From the following species, DNA had been extracted from microfilaria: *Brugia timori* (Dr Tri Baskoro, Indonesia);. *Mansonella (Mansonella) ozzardi* (Prof. Christian Raccurt, Haiti); *Dirofilaria* (*Dirofilaria*) *immitis* (Bayer Animal Health GmbH, Germany).

### Adults

From the following species, DNA had been extracted from adult females: *Acanthocheilonema viteae*, *Brugia malayi* and *Brugia pahangi* (NIAID/NIH Filariasis Research Reagent Resource Center (MTA University of Wisconsin Oshkosh—SJ 770–12); *Loa loa* (Prof Jean Dupouy Camet, Hopital Cochin, France); *Litomosoides sigmodontis* (experimentally infected jirds, *Meriones unguiculatus;* MNHN).

Additional specimens of adult filariae were recovered during dissections of their vertebrate hosts captured in the wild from different geographic areas (Table 1). They were mainly recovered from body cavities and lymphatic vessels or extracted from the dermis, subcutaneous or connective tissue, or tendons of limbs. Identification were made by several experts (OB, KJ, RG, YM, YK, SL, and AR), based on morphological studies. A few species have not yet been named, as their description is still in progress. Samples were fixed and stored in 70% ethanol. The anterior and posterior parts of worms were used for morphological studies, whereas the median part was processed for molecular analysis.

### Ethical statement

Jirds are maintained in the animal facilities of UMR7245, MNHN, as hosts of L. sigmodontis; they are inoculated intraperitoneally with 70 infective larvae and sacrificed 60 days post infection, upon which adult worms are recovered from the peritoneal cavity. All experimental procedures were carried out in strict accordance with the EU Directive 2010/63/UE and the relevant national legislation, namely the French “Décret no 2013–118, 1er février 2013, Ministère de l’Agriculture, de l’Agroalimentaire et de la Forêt”,National licence number 75–1415 approved animal experiments: protocols were approved by the ethical committee of the Museum National d’Histoire Naturelle (Comité Cuvier, Licence: 68–002) and by the “Direction départementale de la cohésion sociale et de la protection des populations” (DDCSPP) (No. C75-05-15).

Some non-human vertebrates were captured for experimental procedures, subject to the ethics approval of the relevant national bodies (S2 Table), while others were obtained at abattoirs or donated to the MNHN by hunters or veterinarians (S2 Table). The MNHN does neither solicit nor compensate for these donations.

Human samples were not collected specifically for this study. They were provided by third parties. Human blood samples were collected with the ethics approval of the relevant national bodies (S2 Table).

The adult female of *Loa loa* had been surgically removed from a male patient in Hospital Cochin, France. The adult subject had given oral consent to Prof J. Dupouy Camet, head of parasitology department at Hospital Cochin, to donate the worm to the MNHN (S2 Table). The MNHN did not and will not compensate for the donation.

### Molecular screening

DNA was extracted using a commercial kit, following manufacturer’s instructions (QIAamp micro kit, Qiagen, Germany). A preliminary step of disruption for two cycles of 30 seconds at 30 Hz using a TissueLyser II (Qiagen, Germany) was added. PCR screening of filarial nematodes was based on seven partial sequences of seven different genes: two mitochondrial genes, 12S rDNA (approximately 450 base-pair (bp) sequence) and cytochrome oxidase subunit I (*coxI*; approximately 600 bp); five nuclear genes, 18S rDNA (approximately 740 bp), 28S rDNA (approximately 900 bp), the myosin heavy chain (*MyoHC*; approximately 785 bp), RNA polymerase II large subunit (*rbp1*; approximately 640 bp), 70 kilodalton heat shock proteins (*hsp70*; approximately 610 bp). Amplification of the 12S rDNA and *coxI* sequences was conducted according to Casiraghi et al. [15]. For the remaining five genes, primer pairs were designed (S3 Table) based on regions that had been found conserved among nine species for which drafts of or complete genome were accessible: from NCBI database *Brugia malayi* (PRJNA27801), *Loa loa* (PRJNA37757), *Onchocerca flexuosa* (Weld, 1856) (PRJEB512), *Dirofilaria immitis* (PRJEB1797) *Onchocerca ochengi* (PRJEB1809); from Nematode Genomes from the Blaxter lab, University of Edinburg (www.nematode.org) *Acanthocheilonema viteae* (nAv.1.0), *Litomosoides sigmodontis* (nLs.2.1); and from Filarial worms Sequencing Project, Broad Institute of Harvard and MIT (http://www.broadinstitute.org/) *Onchocerca volvulus* (Leuckart, 1893) and *Wuchereria bancrofti* (Cobbold, 1877). For each gene, primer pairs adapted to nested PCR were designed (S3 Table). PCRs were processed in a final volume of 20 μl under the conditions summarised in S3 Table. Obtained PCR products were purified using the SV Wizard PCR Purification Kit (Promega, USA) and sequenced directly. A total of 345 sequences were deposited in the GenBank Data Library: KP760116 to KP760460 (S4 Table)

### Phylogenetic analyses

Sequences generated during the current study and previously published sequences from draft/complete genomes were aligned using MAFFT [60]. To check for the absence of stop codons, the alignment of coding genes was translated using EMBOSS Transeq [61], and a comparison with available transcript sequences made. Using the corrected version of the Akaike Information Criterion (AICc), JModelTest analysis [62] was performed to establish the evolution model best adapted to the sequences alignment for each individual gene and for the concatenation of all genes. The General time-reversible plus Invariant sites plus Gamma distributed model (GTR+I+Γ) offered the best fit for *coxI*, *hsp70*, 12S rRNA and 28S rRNA sequences as well as the concatenated alignment; the General Time-Reversible plus Gamma distributed model (GTR+Γ) for *MyoHC* and *rbp1* sequences alignment; and the Kimura 2-parameter Invariant sites plus Gamma distributed model (K80+I+Γ) for 18S rRNA sequences alignment. To root the trees, two species were included as outgroups: *Filaria latala* (Spirurida: Filariidae) and *Protospirura muricola* (Spirurida: Spiruridae). Phylogenetic relationships between onchocercid taxa, based on the concatenated dataset, were performed by Bayesian inference using MrBayes [63]. A partitioned model was implemented to estimate evolution parameters separately for each gene. Two runs were performed using five millions steps with four chains, with tree sampling every 1,000 generations; the first 1,250 points were discarded as burn-in and Posterior Probabilities were calculated from these post-burning trees. In addition, Maximum Likelihood (ML) was used to infer a phylogenetic tree based on the partitioned concatenated dataset, and was executed with 1000 slow bootstrap replicates using RaxML [64]; presented in Supplementary data (S1 Fig).

### Accession numbers

All new sequences generated in this study were deposited in GenBank (http://www.ncbi.nlm.nih.gov/genbank) under the accession numbers: KP760168 to KP760211 for *coxI*, KP760314 to KP760357 for 12S rDNA, KP760116 to KP760167 for 18S rRNA, KP760212 to KP760262 for *MyoHC*, KP760410 to KP760460 for *hsp70*, KP760263 to KP760313 for *rbp1*, KP760358 to KP760409 for 28S rRNA.

## Results/Discussion

### Choosing an outgroup

**Fig 1:**
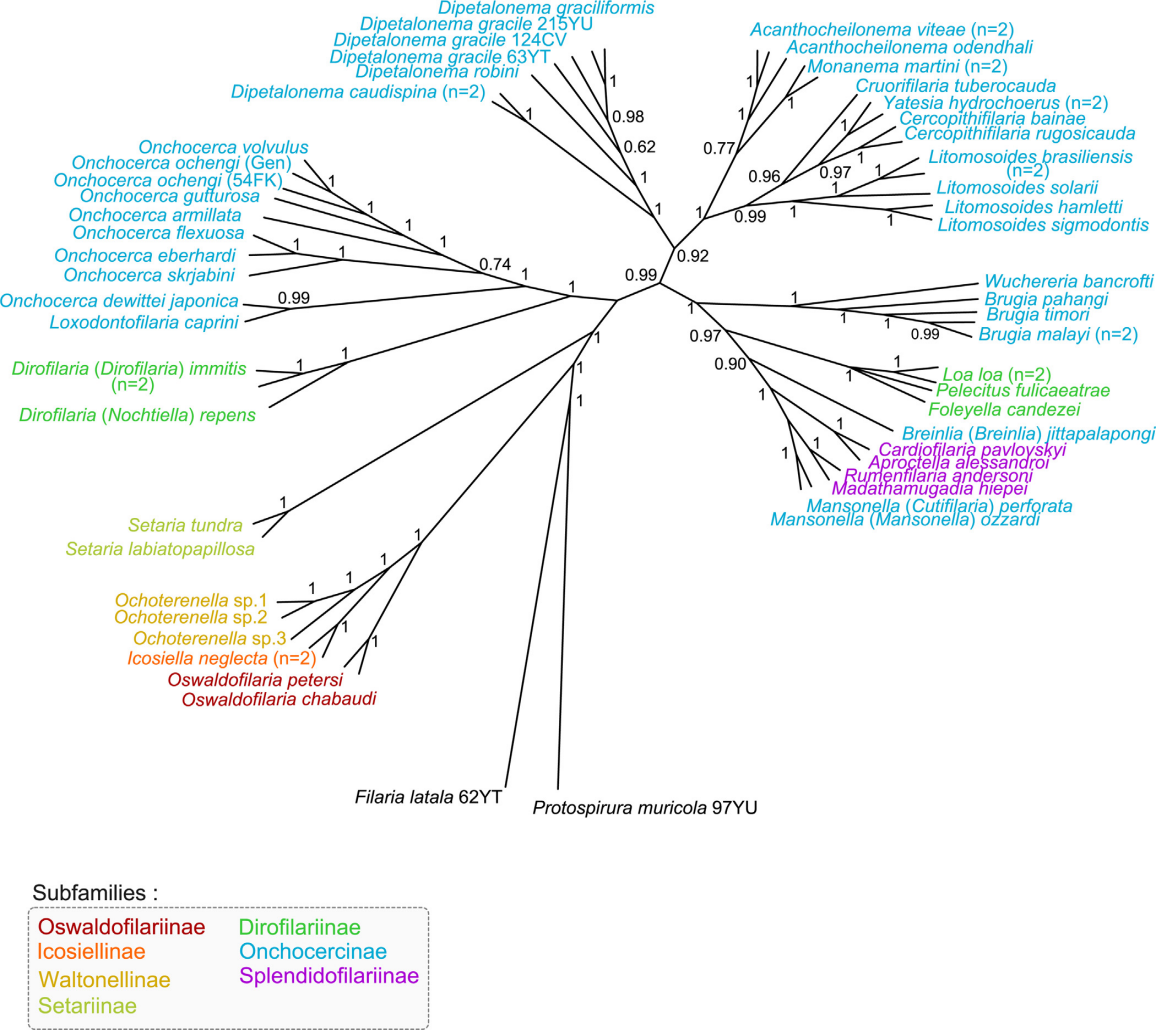
Phylogeny of Onchocercidae based on partitioned concatenated datasets of 12S rDNA, *coxI*, *rbp1*, *hsp70*, *myoHC*, 18S rDNA, and 28S rDNA sequences using Bayesian Inference. The total length of datasets is approximately 4950 bp. Sixty onchocercid specimens (representing 48 species) were analysed. *Filaria latala* and *Protospirura muricola* were used as outgroups. The topology was inferred using Bayesian (B) inference. Nodes are associated with Bayesian posterior probabilities based on one run of 5 million generations. The onchocercid subfamilies are indicated by colour: blue for Onchocercinae, dark green for Dirofilariinae, purple for Splendidofilariinae, pale green for Setariinae, yellow for Waltonellinae, orange for Icosiellinae and red for Oswaldofilariinae.

An important consideration when proposing a comprehensive phylogeny for any taxonomic group is the suitable rooting of the tree and, thus, the choice of appropriate outgroups. Previous studies of the Onchocercidae, based on *coxI* and 12S rRNA gene analyses, have used spirurid representatives of the Thelaziidae [15,16] and Filariidae [16] as outgroups. Furthermore, some phylogenetic analyses of nematodes based on SSU rDNA [21] or complete mitochondrial genome sequences [22] suggested other spirurids as the Physalopteroidea or Diplotriaenoidea to be appropriate outgroups for the Onchocercidae. In the present study, *P*. *muricola* (Spiruridae) and *F*. *latala* (Filariidae) were investigated as potential outgroups. Our multi-gene analyses indicate that this choice is reasonable (Fig 1 and S1 Fig). To support this result, the average genetic divergence between the outgroup and ingroup taxa was about 20% (21% for *P*. *muricola*; 25% for *F*. *latala*), and only about 14% between onchocercid specimens themselves. It was, therefore, concluded that both *P*. *muricola* and *F*. *latala* are appropriate outgroups for the present multi-gene dataset. In congruence with previous systematic analyses [5,6], the chosen rooting clearly supported the monophyly of the Onchocercidae.

### Monophyly of the Onchocercidae

**Fig 2:**
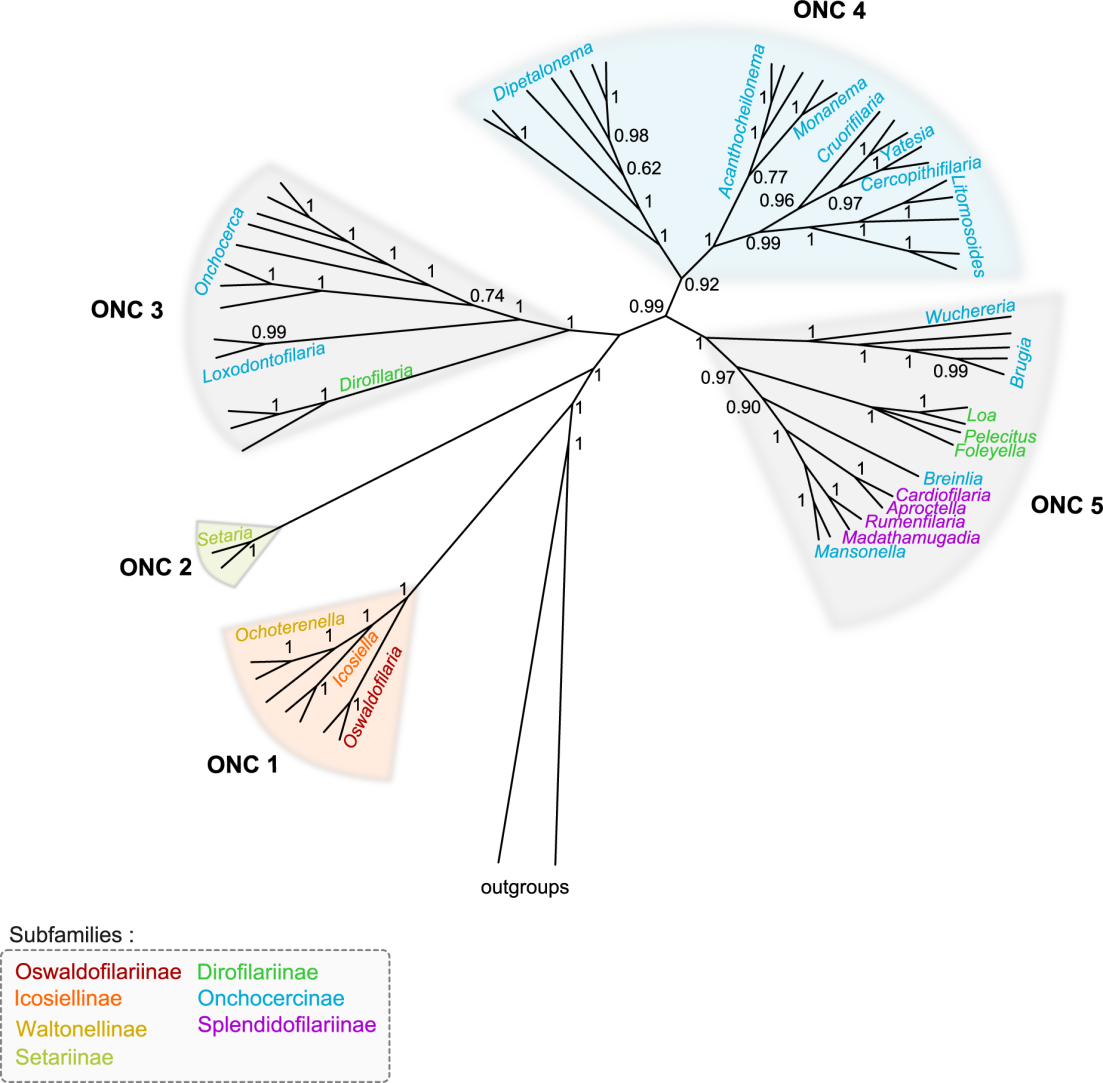
Onchocercid clades based on partitioned concatenated datasets of 12S rDNA, *coxI*, *rbp1*, *hsp70*, *myoHC*, 18S rDNA, and 28S rDNA sequences using Bayesian Inference. The total length of datasets is approximately 4950 bp. Sixty onchocercid specimens (representing 48 species) were analysed. *Filaria latala* and *Protospirura muricola* were used as outgroups. Bayesian posterior probabilities support five clades named ONC1 to ONC5. Dotted areas represented species of the same genus. The topology was inferred using Bayesian (B) inference on one run of 5 million generations. The onchocercid subfamilies are indicated by colour: blue for Onchocercinae, dark green for Dirofilariinae, purple for Splendidofilariinae, pale green for Setariinae, yellow for Waltonellinae, orange for Icosiellinae and red for Oswaldofilariinae.

All onchocercid taxa examined in the present study formed a monophyletic group and five strongly supported clades within the family were identified (Fig 1). Based on their monophyly and to facilitate the description of tree topology, these clades will be referred to as ONC1 to ONC5 (Fig 2).

### The ancestrally derived subfamilies are comforted

In the present phylogenetic analysis, the Oswaldofilariinae, Icosiellinae and Waltonellinae occupied a commun position as a highly supported clade (ONC1) and sister group of all other taxa studied within the Onchocercidae (Fig 1.). The three subfamilies appeared to be closely related, with ONC1 having evolved independently from the remaining onchocercid representatives (Fig 2., S1 Fig). Previous morphological studies revealed a number of ancestral characters in the Icosiellinae and Oswaldofilariinae, such as infective larvae possessing cephalic spines (Icosiellinae), and a vulva that is positioned posterior to the oesophagus (Oswaldofilariinae) [10,65]. Therefore, these subfamilies have traditionally been considered “ancient” [1,10,65]. In addition, it has been suggested that their Gondwanian distribution is an indication of an ancient evolution which occurred prior to the supercontinent’s break-up [1,10]. Interestingly, the Oswaldofilariinae appear to have evolved independently from the remaining two subfamilies of clade ONC1 and were placed as sister group to a clade that consists of *Ochoterenella* spp. (Waltonellinae) and *Icosiella neglecta* (Icosiellinae) (S1 Fig). Indeed, previous studies emphasised the presence of numerous plesiomorphic characters (long oesophagus, large buccal capsule and the presence of deirids) within *Oswaldofilaria* spp. [10]. Moreover, the host spectrum of these subfamilies differs: oswaldofilariines are parasites of squamates, whereas members of Icosiellinae and Waltonellinae are exclusively parasites of anurans [5].

### Setariinae are unusual Onchocercidae with an intermediate position

The systematic position of the Setariinae among the Filarioidea superfamily has long been questioned. Initially the Setariinae had been placed into Filariidae because they share certain morphological similarities with representatives of this family (spines on anterior end of first stage larva and adult cuticular elevation) [11,13]. Nevertheless, based on other biological and morphological traits (*e*.*g*. viviparous, long microfilaria with pointed spineless tail), it would have been equally possible to consign them to the Onchocercidae. At a later stage, the Setariidae was proposed as a family with the purpose of removing the Setariinae from the Filariidae[12], and current classifications list the Setariinae as a subfamily within the Onchocercidae [6]. Such an affiliation between the Setariinae and Onchocercidae has since been supported by many molecular phylogenies [16,19,25,65]. The same was true for the present study, where the two *Setaria* species grouped in one clade (ONC2) with good support (Fig 2). Excepting ONC1, the clade consisting of *Setaria* spp. was sister to all remaining onchocercid taxa sampled in this study. In the past, the Setariinae, Oswaldofilariinae and Waltonellinae were grouped together on the previous phylogenetic trees despite an unresolved topology [16,65]. For some time, morphological studies of infective stages from *Setaria* spp. (long larvae, presence of deirids) have supported the hypothesis that Setariinae may have emerged early in the evolution of Onchocercidae [10,59]. In contrast, the study of larval development of *S*. *labiotopapillosa* suggested that Setariinae, like the remaining Onchocercidae, could have been derived from habronematid ancestors, but that speciation leading to the Setariinae occurred later and independently from the remaining Onchocercidae [1,66]. For example, the development of the glandular cells of the oesophagus in the Setariinae was shown to be closed to the development observed in some potential ancestors of the Onchocercidae, namely the Habronematidae [66,67]. The current phylogeny strongly supported the position of Setariinae as a group derived from an independent speciation to the examined Onchocercidae.

### Subfamilies within the Onchocercidae: A need for systematic revision

The remaining taxa of the Onchocercidae analysed in this study grouped in three distinct and well supported clades (ONC3, ONC4 and ONC5) (Fig 2), including representatives of the Dirofilariinae, Onchocercinae and Splendidofilariinae. It is noteworthy that the tree topology demonstrated here is not congruent with classic systematic delineations:

- The first clade, ONC3 (Fig 2), contained representatives of the genera *Dirofilaria* (Dirofilariinae), *Loxodontofilaria* and *Onchocerca* (Onchocercinae) as suggested by several earlier molecular analyses [14,16,18,25]. Although these genera belong to two different subfamilies when looking at established classifications [13,68], a possible need to merge these subfamilies is reinforced by several biological traits [65]. Firstly, the infective stages of *Dirofilaria* spp. and *Onchocerca* spp. possess morphological similarities (normally developed buccal capsule; cylindrical tail with tiny caudal lappets) [59,65]. Secondly, there are shared life history traits: an early first moult in the vertebrate host takes place early, *i*.*e* 2–3 days postinfection, in *Dirofilaria* spp. and *Onchocerca* spp. [1], as opposed to a late moult, *i*.*e*. 6–10 days postinfection, in all other onchocercid species studied thus far [68]. No information is currently available on the infective larvae and development in the final host of representatives of *Loxodontofilaria*. This clade (ONC3) (Fig 2) constitutes the most ancestrally derived group belonging to modern Onchocercidae. Clade ONC3 was sister group of both clades ONC4 and ONC5 (Fig 2), suggesting an independent evolution.
- The second clade, ONC4 (Fig 2), was composed of the genera *Dipetalonema*, *Acanthocheilonema*, *Monanema*, *Cruorifilaria*, *Cercopithifilaria*, *Yatesia* and *Litomosoides*. For a long time, taxonomists used *Dipetalonema* as a “catch all” genus [69]. In years to follow, numerous subgenera formerly included in this genus were elevated to generic rank, whereas other species were transferred in newly erected genera; together, they were referred to as the “*Dipetalonema* lineage” [69,70]. This lineage combined those species of the Onchocercinae that present adults with a long tail, a buccal capsule divided into two segments (or three in *Skrjabinofilaria* spp.), and a caudal extremity with two subterminal lappets [70]. These structures might, however, be more or less atrophied in the more specialised forms, such as seen in the buccal capsule of *Monanema* and *Cercopithifilaria* species [69,71]. The present phylogenetic tree supports the accuracy of the *Dipetalonema* lineage, as all genera included in the clade ONC4, with the exception of *Litomosoides*, have been assigned to this lineage [69,70]. Prior to this study, molecular analyses had only supported the clustering of *Dipetalonema* with *Acanthocheilonema* [16]. A closer look at clade ONC4 revealed its division into two groups (Fig 2):
  i) *Dipetalonema* spp. were sister to all remaining taxa within clade ONC4 (Fig 2). This position agrees with earlier hypotheses that, based on ancestral morphological particularities (divided oesophagus, well developed and highly cuticularized buccal capsule) the genus *Dipetalonema* should be considered distinct from the remaining genera in the *Dipetalonema* lineage [70]. Moreover, it had been proposed that the genus may have been derived through geographic isolation in the Neotropical region after the Gondwana fragmentation and so would be a paleoendemic South American genus [10,70].
  ii)The second subclade group comprised members of *Acanthocheilonema*, *Monanema Cercopithifilaria*, *Yatesia*, *Cruorifilaria* and *Litomosoides* seems more derived. However, species of *Acanthocheilonema* and *Monanema* seemed to be more closely related and were sister to the remaining taxa in this group. Earlier taxonomic studies described *Acanthocheilonema* as an ancestral genus due to several plesiomorph characters of its representatives (strongly divided oesophagus, highly cuticularized buccal capsule, shape of right spicule of male) [10,65,70]. In contrast, the morphology of *Monanem*a includes both ancestral (numerous caudal papillae distributed along length of tail) as well as derived characters (undivided oesophagus) [69,71]. Bain *et al*. (70) considered *Monanema* as derived from Ethiopian *Acanthocheilonema* species [70]. The remaining genera formed two sister groups: the one uniting taxa of *Cruorifilaria*, *Yatesia* and *Cercopithifilaria*, the other containing species of *Litomosoides*. Earlier studies had suggested *Cercopithifilaria* to be derived from *Acanthocheilonema* and, as indicated by the derived morphological characters of its members (reduced buccal capsule, undivided oesophagus, shape of right spicule of male, number and repartition of caudal papillae), to represent the most evolved forms of the *Dipetalonema* lineage [10,70]. *Litomosoides* had not been included in the *Dipetalonema* lineage in the 1976 classification by Chabaud and Bain [69]. The latter authors did, however, point out that the reason for this omission was somewhat arbitrary. In fact, *Litomosoides* species do share morphological similarities with other representatives of the *Dipetalonema* lineage (long tail, long buccal capsule) [6,14,69].
- The third clade (ONC5) united genera belonging to the Dirofilariinae (*Loa*, *Foleyella*, *Pelecitus*), Onchocercinae (*Wuchereria*, *Brugia*, *Breinlia*, *Mansonella*) and Splendidofilariinae (*Aproctella*, *Cardiofilaria*, *Madathamugadia*, *Rumenfilaria*) (Fig 2). Most human filariae were included in this clade, with the exception of *O*. *volvulus*, which groups in clade ONC3. Three groups could be pinpointed in clade ONC5:
  i) The first group, including *Brugia* and *Wuchereria*, was sister to all remaining representatives of clade ONC5. Previous molecular studies highlighted a close relation between these two genera [14,16,25,65], which is supported by shared morphological similarities (reduced buccal capsule, glandular part of oesophagus, vagina with bends and chamber in adults; infective larvae with a long tail and three lappets) [1,72]. Moreover, their mode of transmission and development is similar (culicid vector, lymphatic location in the host) [9].
  ii) The second group comprised species exclusively assigned to the Dirofilariinae: *L*. *loa*, *P*. *fulicaeatrae* and *F*. *candezei*. These parasites did not group together in previous molecular analyses [16,19]. However, they share morphological similarities, based on which this subfamily had originally been introduced (highly developed caudal alae of adult males) [9,12]. Futhermore, *L*. *loa* and *Pelecitus scapiceps* Leidy, 1886 present similar biological traits, such as a late third moult (respectively on day 8 and day 6 postinfection) and a fourth moultprior to day 20 postinfection, both of which are close to the developmental pattern seen in many species of the Onchocercinae and Splendidofilariinae [31,68].
  iii) The last group in clade ONC5 was composed of representatives of Splendidofilariinae and Onchocercinae. Up to now, Splendidofilariinae have rarely been studied [19,25]. In the present analysis, species of *Madathamugadia* and *Rumenfilaria* (Splendidofilariinae) appeared more closely related to the two species of *Mansonella* (Onchocercinae), of which they were a sister group, than they were to the remaining splendidofilariine genera, *Cardiofilaria* and *Aproctella* species. The close relationship between *Cardiofilaria* and *Aproctella* has been underlined by morphological similarities in infective larvae (body length exceeding 1300μm, thick and short tail, ending in two small lateral terminal papillae) [33]. *Mansonella* is a complex and taxonomically challenging genus and numerous attempts to reorganise its classification, including description of several subgenera, have been made throughout the years [1,69,73]. Its relationship with representatives of the Splendidofilariinae has not been resolved, but it does share similarities with some splendidofilariine genera such as the *Madathamugadia* (*e*.*g* short and thin infective larvae with long tail) [35,59]. Species of *Breinlia* have been described as Australian representatives of the *Dipetalonema* lineage which have more recently migrated into Asia, and are morphologically distinct from the remaining species of this lineage [70]. Their somewhat isolated position was upheld by the current phylogeny. It is interesting to note that these various species (*e*.*g*. *Madathamugadia* or *Foleyella*) have been described as an evolved group [74].

One of the main difficulties in comparing the results of the present study with those of earlier molecular analyses was either the lack of broad taxonomic sampling or incompletely resolved deeper phylogenetic relationships in preceding studies [15,16,18,19]. However, an equivalent to clade ONC5 clade had been identified before,in the first phylogeny including *L*. *loa*, *B*. *malayi* and *W*. *bancrofti* and *Mansonella* (*M*.) *perstans* (Manson, 1891) [14]. A similar clade, including *B*. *malayi*, *W*. *bancrofti*, *L*. *loa* and *Chanderella quiscali* Linstow, 1904, a Splendidofilariinae, was confirmed by a recent analysis of mitochondrial genomes [25].

To summarise, the present phylogeny did neither support the monophyly of the Dirofilariinae, Onchocercinae nor Splendidofilariinae (see ONC3 and ONC5, Fig 2). Interestingly, however, the relationships exhibited between the various taxa in clades ONC3 and ONC5, although traditionally included in different subfamilies, are supported by morphological as well as biological features (see discussion above). Based on the combined analysis of molecular, structural and developmental characters, we, thus, conclude that a reassessment of the boundaries between these three subfamilies is called for.

### Evolution of filariae, their vector and host: Is there a relationship?

**Fig 3:**
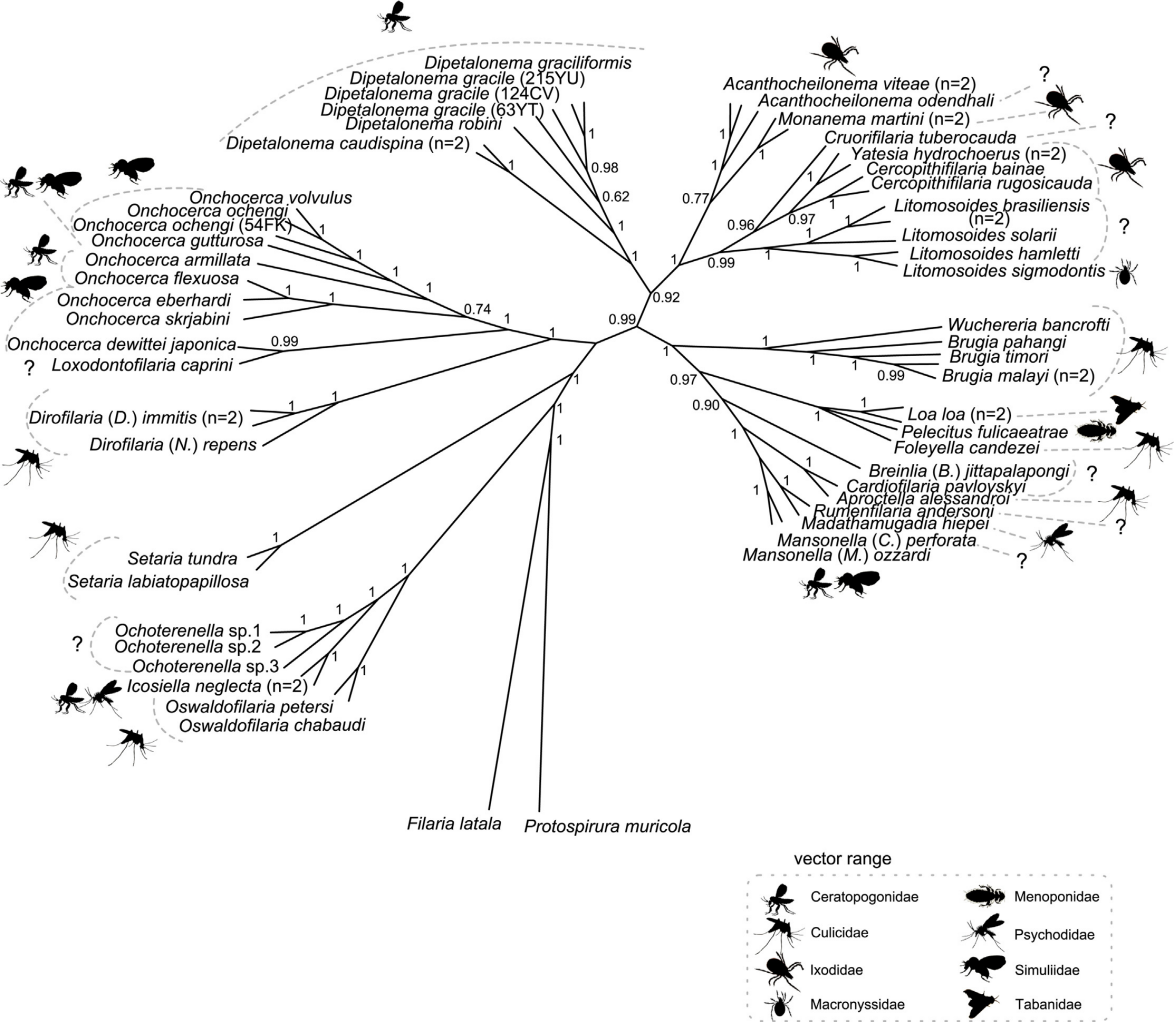
Phylogeny of Onchocercidae based on partitioned concatenated datasets of 12S rDNA, *coxI*, *rbp1*, *hsp70*, *myoHC*, 18S rDNA, and 28S rDNA sequences using Bayesian Inference with indication of vector range. The total length of datasets is approximately 4950 bp. Sixty onchocercid specimens (representing 48 species) were analysed. *Filaria latala* and *Protospirura muricola* were used as outgroups. The topology was inferred using Bayesian (B) inference. Nodes are associated with Bayesian posterior probabilities based on one run of 5 million generations. The vector family for each filariae species is indicated to the right of the tree using the specified symbols.

**Fig 4:**
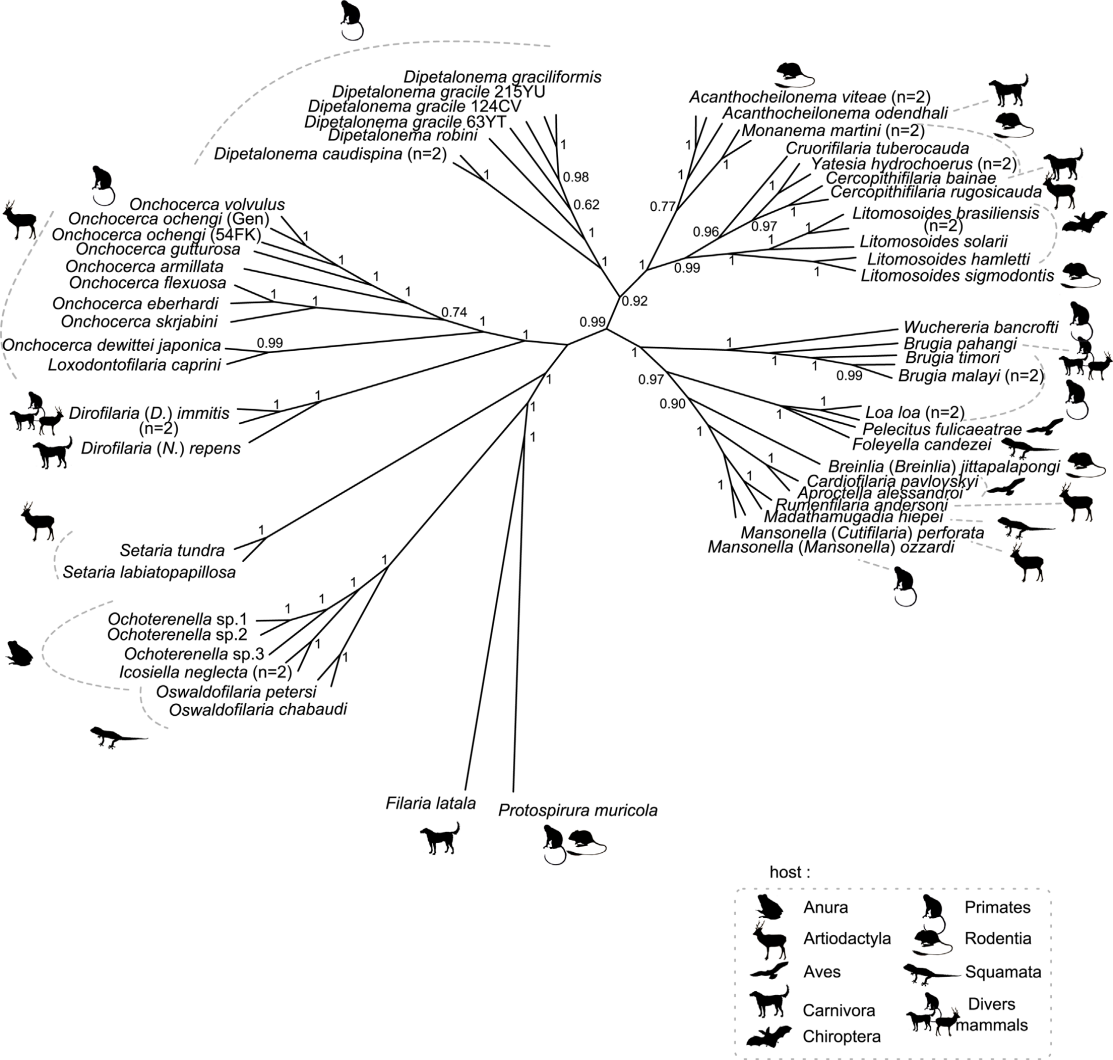
Phylogeny of Onchocercidae based on partitioned concatenated datasets of 12S rDNA, *coxI*, *rbp1*, *hsp70*, *myoHC*, 18S rDNA, and 28S rDNA sequences using Bayesian Inference with indication of host vertebrate range. The total length of datasets is approximately 4950 bp. Sixty onchocercid specimens (representing 48 species) were analysed. *Filaria latala* and *Protospirura muricola* were used as outgroups. The topology was inferred using Bayesian (B) inference. Nodes are associated with Bayesian posterior probabilities based on one run of 5 million generations. The host vertebrate order for each filariae species is indicated to the right of the tree using the specified symbols.

The parasitic life style of the Onchocercidae has led to the reduction of certain morphological traits and promoted convergent evolution in others [10], reducing the usefulness of structural characters in developing a phylogenetic framework for this family. One might thus look at the role of vectors and hosts for further clues as to the speciation of the Onchocercidae. Three (ONC1, ONC2 and ONC3) of the five clades exhibited on the current phylogenetic tree use exclusively dipterans as vectors. In addition, dipterans are involved in the transmission of members of clades ONC4 and ONC5, supporting a hypothesis that old evolutionary bonds exist between this order and the Onchocercidae (Fig 3, S1 Table). The diversity of vectors used in clades ONC4 and ONC5 (Fig 3), on the other hand, suggests that the evolution of filarial nematodes is not strictly constrained by their vector but does permit changes to other, more appropriate groups of vectors, to ensure parasite transmission, colonisation of new ecological niches and diversification. Clade ONC4 shows an adaptation to a new group of vectors–ticks and mites. It should, however, be emphasised that some *Acanthocheilonema* spp. (ONC4) are known to use fleas, hippoboscid flies or sucking lice as vectors [9], and *Dipetalonema* spp. are transmitted by ceratopogonid biting midges (Fig 3). Considering relationships of the Onchocercidae with their definitive hosts, the present analysis revealed a group of parasites adapted exclusively to cold blooded tetrapods, namely squamates, crocodilians and amphibians (ONC1), while their sister group diversified in various groups of mammals (ONC2-5), as well as birds and squamates (ONC5) (Fig 4). It has been suggested that the parasite-host relationships of *M*. *hiepei* (parasite of the gecko *Chondrodactylus turneri*) and *F*. *candezei* (parasite of lizards) may be the result of secondary capture with the narrow geographical localisation of *Foleyella* and *Madathamugadia*, *i*.*e*. Africa and Madagascar, supporting the hypothesis of capture phenomena [74]. The present phylogenetic analysis does not support a close relationship between parasites of birds, but representation of avian parasites was limited. All three parasites of birds formed part of clade ONC5, which presented the most heterogeneous host range within the Onchocercidae, comprising parasites of three classes of vertebrates, reptiles, birds and mammals. The diverse mammalian hosts represented in clades ONC3-5 indicate multiple events of host-switching and radiation within the evolutionary history of the group. In summary, the overall picture revealed by the current analysis does not suggest a broad pattern of onchocercid parasite-host/vector coevolution and is consistent with earlier studies suggesting that parasitic nematode speciation could be related to a variety of events of host switching and host acquisition and/or geographical and ecological drift [75–77].

### Concluding remarks

The present study casts doubt on the validity of of the currently eight traditional onchocercid subfamilies. Traditional classifications of the Onchocercidae have been established mainly on the basis of morphological characters of adult worms. However, as suggested by previous authors [10,68], biological features, such as larval biology or the morphology of the third-stage larva, may offer a better phylogenetic resolution. It is hoped that an approach combining broad molecular taxonomic sampling and traditional morphological as well as life history studies, will eventually lead to the development of more comprehensive phylogenetic hypotheses for this fascinating group of nematodes.

## Supporting Information

S1 Fig Phylogeny of Onchocercidae based on partitioned concatenated datasets of 12S rDNA, *coxI*, *rbp1*, *hsp70*, *myoHC*, 18S rDNA, and 28S rDNA sequences using Maximum Likelihood Inference. The total length of datasets is approximately 4950 bp. Sixty onchocercid specimens (representing 48 species) were analysed. *Filaria latala* and *Protospirura muricola* were used as outgroups. The topology was inferred using Maximum Likelihood (ML) inference. Nodes are associated with Bootstrap values based on 1000 replicates. The onchocercid subfamilies are indicated by colour: blue for Onchocercinae, dark green for Dirofilariinae, purple for Splendidofilariinae, pale green for Setariinae, yellow for Waltonellinae, orange for Icosiellinae and red for Oswaldofilariinae.
(TIF)

S1 Table Supplementary information of filarial species used in phylogenetic analyses in this study. The species were classified by subfamilies indicated in the first column. The name of the species as well as the author(s) and date are given in the second column. Columns 3 to 8 present species information, i.e. family host, order host, localisation in the host, genera vector, family vector and geographical distribution. The references of morphological studies on which species identifications were based are listed in the last column. For the most part of the sampling some supplementary information were based on Anderson et *al*. [9].
(XLSX)

S2 Table Ethical statement on vertebrate hosts and human samples. Vertebrate host are indicated in the first column and their collection place is given in the second column. The name of the collected filarial species is given in the third column. Columns 4 and 5 present ethical information on the host collection, *i*.*e*. the capture permit number and the ethical committee or legal entity for scientific procedures; Columns 6 to 9 describe non-scientific procedures in which filariae are recovered from vertebrates after hunting, necropsies in slaughter houses, or post-veterinary or post-medical procedures. The number of MNHN collection registration is listed in the last column.
(XLS)

S3 Table Primers and PCR programs used in this study. Abbreviations: Step 1: denaturation; Step 2: annealing; Step 3: elongation; T: temperature (°C); D: duration (sec); N: number of cycles. * Indicates the primers designed for nested PCR. References: PS means newly design for the present study.
(XLS)

S4 Table Accession number list of filarial species and outgroups used for phylogenetic analyses. The name of the species are given in the first column. The identification number of samples are given in the second column. Columns 3 to 9 present accession number for each analysed genes, i.e. *coxI* (cytochrome oxidase subunit I), 12S rRNA, 18S rRNA, *MyoHC* (myosin heavy chain), *hsp70* (70 kilodalton heat shock proteins), *rbp1* (RNA polymerase II large subunit) and 28S rRNA. Abbreviations: ext. indicated external samples.
(XLSX)

S1 Appendix List of author(s) and date associated with name of the vertebrate hosts or vector species. (DOCX)
