# Supplementary figures and images for: Shaking the Tree: Multi-locus Sequence Typing Usurps Current Onchocercid (Filarial Nematode) Phylogeny
Source: PLoS Negl Trop Dis. 2015 Nov 20;9(11):e0004233. doi: 10.1371/journal.pntd.0004233 (PMC4654488; doi:10.1371/journal.pntd.0004233)

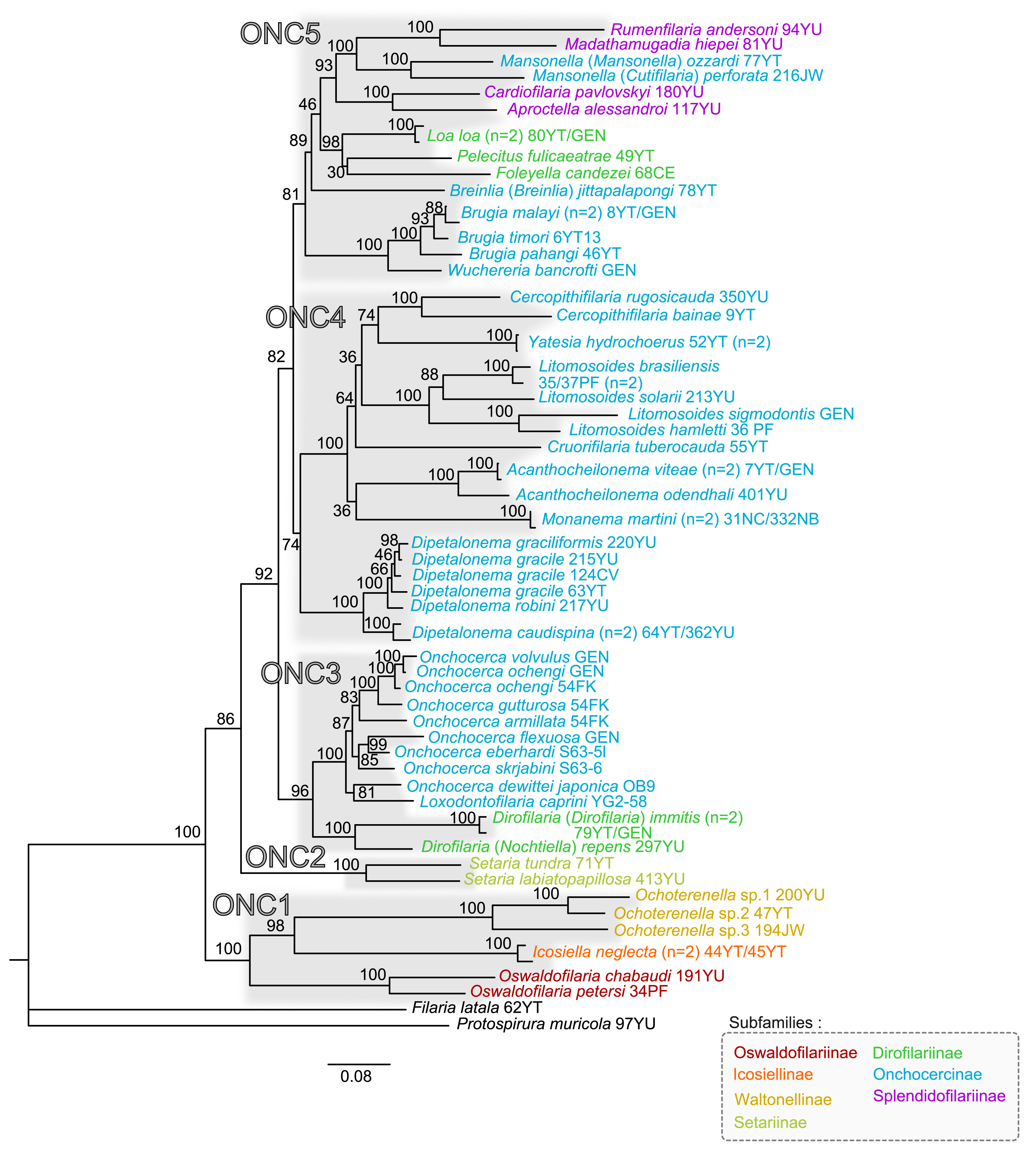

Supplement: S1 Fig — The total length of datasets is approximately 4950 bp. Sixty onchocercid specimens (representing 48 species) were analysed. Filaria latala and Protospirura muricola were used as outgroups. The topology was inferred using Maximum Likelihood (ML) inference. Nodes are associated with Bootstrap values based on 1000 replicates. The onchocercid subfamilies are indicated by colour: blue for Onchocercinae, dark green for Dirofilariinae, purple for Splendidofilariinae, pale green for Setariinae, yellow for Waltonellinae, orange for Icosiellinae and red for Oswaldofilariinae. (TIF) [file pntd.0004233.s001.tif]
