# Supplementary material for: Shaking the Tree: Multi-locus Sequence Typing Usurps Current Onchocercid (Filarial Nematode) Phylogeny
Source: PLoS Negl Trop Dis. 2015 Nov 20;9(11):e0004233. doi: 10.1371/journal.pntd.0004233 (PMC4654488; doi:10.1371/journal.pntd.0004233)
Supplement: S1 Appendix — (DOCX) [file pntd.0004233.s006.docx]

*Agama agama* (Linnaeus, 1758)

*Arvicanthis niloticus* (Geoffroy,1803)

*Ateles* E. Geoffroy, 1806

*Ateles paniscus* (Linnaeus, 1758)

*Bos taurus* Linnaeus, 1758

*Callorhinus ursinus* (Linnaeus, 1758)

*Canis familiaris* Linnaeus, 1758

*Capreolus capreolus* (Linnaeus, 1758)

*Carollia perspicillata* (Linnaeus, 1758)

*Cebus apella* (Linnaeus, 1758)

*Cebus olivaceus* Schomburgk, 1848

*Cervus nippon* Temminck, 1838

*Chondrodactylus turneri* (Gray, 1864)

*Crocodilurus amazonicus* (spix, 1825)

*Glossophaga soricina* Pallas, 1766

*Gorilla gorilla* Savage, 1847

*Homo sapiens* Linnaeus, 1758

*Hydrochoerus hydrochaeris* (Linnaeus, 1766)

*Ixodes ricinus* (Linnaeus, 1758)

*Lagothrix* *poeppigii* Schinz, 1844

*Meriones unguiculatus* (Milne-Edwards, 1867)

*Naemorhedus crispus* (Robert Swinhoe, 1870)

*Oriolus oriolus* (Linnaeus, 1758)

*Panthera leo* (Linnaeus, 1758)

*Pelophylax kl. esculentus* (Linnaeus, 1758) (syn. *Rana esculenta)*

*Pelophylax ridibundu* (Pallas ,1771) (syn. *Rana ridibunda)*

*Phyllomedusa bicolor* (Boddaert, 1772)

*Podiceps nigricollis*, Brehm, 1831

*Rangifer tarandus* (Linnaeus, 1758)

*Rattus tanezumi* Temminck, 1844

*Rhinella granulosa* (Spix, 1824)

*Rhinella marina* (Linnaeus, 1758)

*Saimiri sciureus* (Linnaeus, 1758)

*Saltator similis* D'Orbigny & Lafresnaye, 1837

*Sus scrofa leucomystax* Temminck, 1842

*Trachops cirrhosis* (Spix, 1823)

*Tropidurus torquatus* (Wied-Neuwied, 1820)
